# Early Sepsis Detection in Adult Patients with Suspected Sepsis in an Emergency Setting: A Sequential Strategy of Monocyte Distribution Width and Presepsin

**DOI:** 10.3390/diagnostics15202574

**Published:** 2025-10-13

**Authors:** Hanah Kim, Mina Hur, Hyejung Lee, Gun-Hyuk Lee, Kyeong Ryong Lee, Ferdinando Mannello

**Affiliations:** 1 Department of Laboratory Medicine, Konkuk University School of Medicine, Seoul 05030, Republic of Korea; md.hkim@gmail.com (H.K.); applemint_jr@naver.com (H.L.); 2 Department of Laboratory Medicine, Wonkwang University School of Medicine, Iksan 54538, Republic of Korea; leegunhyuk93@gmail.com; 3 Department of Emergency Medicine, Konkuk University School of Medicine, Seoul 05030, Republic of Korea; 20020001@kuh.ac.kr; 4 Unit of Clinical Biochemistry, Section of Biochemistry and Biotechnology, Department of Biomolecular Sciences, University of Urbino Carlo Bo, 61029 Urbino, Italy; ferdinando.mannello@uniurb.it

**Keywords:** sepsis, monocyte distribution width, presepsin, emergency department, early detection, sequential strategy

## Abstract

**Background/Objectives**: Monocyte distribution width (MDW) is a US FDA-cleared early sepsis indicator for adult patients presenting to the emergency department (ED). Presepsin, a soluble CD14 subtype, is another sepsis biomarker reflecting innate immune activation. We explored the clinical utility of sequential MDW and presepsin testing for early sepsis detection in the ED. **Methods**: In a total of 281 adult ED patients with suspected sepsis (including 128 patients with confirmed sepsis), MDW was measured on a DxH 900 analyzer (Beckman Coulter, USA), and presepsin level was measured using the HISCL Presepsin assay (Sysmex, Japan). Diagnostic performances of MDW, presepsin, and their combination (MDW followed by presepsin) were compared using sensitivity, specificity, and area under the curves (AUC) of receiver operating characteristic (ROC) curve analyses. **Results**: MDW, presepsin, and their combination were comparable for diagnosing sepsis (AUC ranges: 0.52–0.65). Compared with MDW and presepsin, their combination increased diagnostic sensitivity (90.6%, 89.8%, and 98.4%, respectively). Moreover, the sequential strategy significantly reduced false-negative results compared to each biomarker (2 [1.6%] for the sequential strategy vs. 12 [9.4%] for MDW vs. 13 [10.2%] for presepsin, *p* < 0.001). **Conclusions**: Compared with individual measurement of MDW and presepsin, the sequential strategy of MDW followed by presepsin would improve early sepsis detection in ED patients by significantly reducing false negatives. This approach would ensure timely and effective triage for ruling in septic patients, potentially leading to improved patient outcomes.

## 1. Introduction

Sepsis is a life-threatening response to infection that requires rapid identification and immediate treatment to prevent adverse clinical outcomes [1,2,3]. Although the sequential organ failure assessment (SOFA) scoring system is an essential tool for diagnosing sepsis in the Sepsis-3 criteria, it is often impractical in real-time clinical practice, especially in the emergency department (ED) [1,4]. As an alternative, the quick SOFA (qSOFA) score has been introduced for detecting sepsis to prompt further evaluation and treatment; however, its diagnostic and prognostic accuracy is limited, highlighting the unmet need for more effective diagnostic strategies [1,2,5,6,7,8,9].

As a response to the signals of infection or tissue injury, monocytes may undergo an inflammatory form of cell death, with their size and morphology being changed [10,11]. Monocyte distribution width (MDW) may quantify this size variability and is automatically generated as a part of cell population data in the complete blood count (CBC) by DxH 800 and DxH 900 hematology analyzers (Beckman Coulter, Miami, FL, USA) [12,13]. MDW has been cleared by the US Food and Drug Administration (FDA) as an early sepsis indicator for adult patients presenting to the ED [13]. Its clinical cut-off has been suggested to be 20.0 for whole blood samples in di-potassium ethylenediaminetetraacetic acid (K_2_-EDTA) and 21.5 for whole blood samples in tri-potassium EDTA (K_3_-EDTA) [14].

Presepsin is a monocyte-derived soluble subtype of CD14 (sCD14-ST), specifically a 13 kDa N-terminal fragment produced by cathepsin D. It plays a key role in activating the innate immune response and has emerged as a biomarker for the early diagnosis and monitoring of sepsis [15]. Presepsin levels may increase in response to bacterial infections and decrease following effective treatment [15,16,17]. Previous studies demonstrated its utility not only for early diagnosis but also for prognosis prediction in sepsis [18,19,20,21].

While the utilities of MDW and presepsin for diagnosing sepsis have been studied individually, to our knowledge, their combined use has never been explored in real-world ED settings. In this study, we wanted to evaluate the diagnostic utility of these two sepsis biomarkers, MDW and presepsin, in ED patients with suspected sepsis. Considering that they are both monocyte-related biomarkers, we questioned how different their diagnostic performance would be as a stand-alone test and how they can be combined into an effective strategy to triage ED patients with suspected sepsis. We hypothesized that, if automatically generated MDW is used as an initial testing and then presepsin is added as a reflex testing for patients with normal MDW, such a sequential application would be an effective rule-in strategy to ensure the inclusion of all critically ill septic patients. We also explored which MDW cut-off would be optimal to maximize early detection of sepsis while minimizing missed sepsis cases.

## 2. Materials and Methods

### 2.1. Study Population

Between June 2020 and June 2021, a total of 481 patients were consecutively recruited at Konkuk University Medical Center (KUMC). They were all adult patients (>19 years of age) who presented to the ED with clinical suspicion of sepsis, and in all patients, routine laboratory tests were performed, including CBC, C-reactive protein (CRP), procalcitonin (PCT), etc. [22]. Excluding 200 patients with inadequate residual serum samples (volume < 1 mL, hemolysis, or clotting), 281 patients were finally included in this study.

In this study population, 128 patients (45.6%) were confirmed as having sepsis (including 14 patients with septic shock) according to the Sepsis-3 criteria, and the remaining 153 patients (54.4%) were considered as having non-sepsis [1]. There were no patients with Coronavirus Disease 2019 (COVID-19), which could have influenced clinical outcomes [23]. Their medical records were reviewed retrospectively to retrieve demographic, clinical, and laboratory data (Table 1).

In this cross-sectional, in vitro evaluation study, residual samples were used for the measurement of presepsin level, and patients’ records were analyzed retrospectively. The study protocol was approved by the Institutional Review Board of KUMC (approval No. KUMC 2025-02-010). Due to the retrospective study design, the use of anonymized clinical data, and the absence of additional sampling or interventions, the requirement for obtaining written informed consent from the study population was waived.

### 2.2. Measurement of MDW and Presepsin

In all patients, samples were collected in K_3_-EDTA vacutainers at presentation to the ED, and CBC was measured using a DxH 900 within two hours of collection. MDW values were generated automatically as a part of the CBC, and the manufacturer-suggested MDW cut-off was 21.5 [14]. The DxH 900 integrates volume (V), conductivity (C), and multi-angle light scatter (Sn) measurements, collectively known as VCSn technology, to assess morphological changes in white blood cells (WBCs) [24]. Monocytes are identified on the volume versus rotated light scatter data plot, where volume corresponds to cell size [14]. The MDW is calculated as the standard deviation (SD) of monocyte volume values, representing the dispersion of the monocyte population derived from the WBC differential data plot [14]. All maintenance, operational functions, and calibration were performed according to the manufacturer’s instructions. A quality control check was performed using Coulter 6C Cell Controls (Beckman Coulter) at three concentration levels, with analyses conducted using software version 1.0.0.

Residual sera were stored at −70 °C, and the frozen samples were thawed at room temperature and gently mixed immediately before analysis. Presepsin level was measured using an in vitro diagnostic assay, the HISCL Presepsin assay (Sysmex, Kobe, Japan), on an HISCL 5000 analyzer (Sysmex). It is a delayed one-step sandwich chemiluminescence enzyme immunoassay, with a measurement range of 20 to 30,000 pg/mL [25,26]. The reference interval with 95th percentile is 333 pg/mL, and a manufacturer-recommended cut-off of 500 pg/mL is indicative of suspected sepsis [25,27]. All procedures were conducted according to the manufacturer’s instructions.

### 2.3. Statistical Analysis

Continuous variables were presented as medians with interquartile ranges (IQRs), and categorical variables were presented as numbers with percentages. The Kolmogorov–Smirnov test was used to check the normality of data distribution. Grubb’s test was used to detect potential statistical outliers, and there was no significant outlier. For the comparison between the non-sepsis and sepsis groups, the Mann–Whitney U test was used for continuous variables, and the chi-squared test was used for categorical variables.

MDW and presepsin levels were divided into quartiles, and we assessed the associations of Q1 vs. Q2–Q4 of each biomarker with sepsis stratified by qSOFA (<2 vs. ≥2). For each stratum, we calculated odds ratios (ORs) with 95% confidence intervals (CIs). Homogeneity of stratum-specific ORs was tested with the Breslow–Day (Tarone-adjusted) test. When no heterogeneity was detected, a common OR was estimated using the Cochran–Mantel–Haenszel (CMH) method and tested with the CMH chi-square [28].

To evaluate the relationship between MDW and presepsin, presepsin levels were log-transformed to mitigate skewness, approximate normality, and stabilize variance. We computed Pearson’s correlation between MDW and log-transformed presepsin and interpreted correlation coefficients (r) with 95% CI as follows: <0.30, negligible; 0.30–0.50, low; 0.50–0.70, moderate; 0.70–0.90, high; 0.90–1.00, very high [29]. Patients were further divided into quadrants using the cut-offs (MDW, 21.5; presepsin, 500 pg/mL), and the proportion of septic patients was compared across quadrants using the chi-squared test.

The diagnostic performance of MDW (with cut-off of 21.5), presepsin, and their combination was compared using sensitivity, specificity, and the area under the curve (AUC) of the receiver operating characteristic (ROC) curve with respective 95% CI. The data-derived optimal cut-off of MDW was identified using the Youden index. ROC curves were additionally used to summarize sensitivity, specificity, and AUC for MDW at three cut-offs (rule-in, manufacturer-suggested, and data-derived optimal cut-offs). To assess the sequential strategy, patients were grouped by MDW cut-offs (rule-in and manufacturer-suggested cut-offs) and by presepsin > 500 pg/mL. All data were analyzed using MedCalc Statistical Software (version 20.201, MedCalc Software Ltd., Ostend, Belgium). Rounding rules were applied to summary statistics [30], and a *p*-value < 0.05 was considered statistically significant.

## 3. Results

The median value of MDW was significantly higher in the sepsis group than in the non-sepsis group: 26.5 (IQR, 25.6–27.6) vs. 24.5 (23.9–25.2), *p* = 0.0014. The median presepsin level was also significantly higher in the sepsis group than in the non-sepsis group: 1384 pg/mL (1113–1697) vs. 590 pg/mL (519–730), *p* < 0.001 (Table 1). MDW and presepsin level were divided into quartiles (MDW: Q1, 18.7–22.8; Q2, 22.9–25.2; Q3, 25.3–29.2; Q4, 29.3–57.2; presepsin: Q1, 161–494 pg/mL; Q2, 495–878 pg/mL; Q3, 879–1660 pg/mL; Q4, 1660–30,000 pg/mL). In the analyses stratified by qSOFA (<2 vs. ≥2), MDW Q2–Q4 (vs. Q1) showed higher odds of sepsis in both strata (qSOFA < 2: OR 4.2 [95% CI 1.4–12.8]; qSOFA ≥ 2: OR 8.3 [4.3–15.9]). The common OR was 7.0 (4.0–12.2; *p* < 0.001), with no evidence of heterogeneity (Breslow–Day test, *p* = 0.307). Similarly, presepsin Q2–Q4 (vs. Q1) showed higher odds of sepsis in both strata (qSOFA < 2: OR 18.3 [4.2–79.5]; qSOFA ≥ 2: OR 5.24 [2.78–9.86]). The common OR was 6.2 (3.4–11.0; *p* < 0.001), with no evidence of heterogeneity (Breslow–Day test, *p* = 0.117) (Table 2).

**Figure 1:**
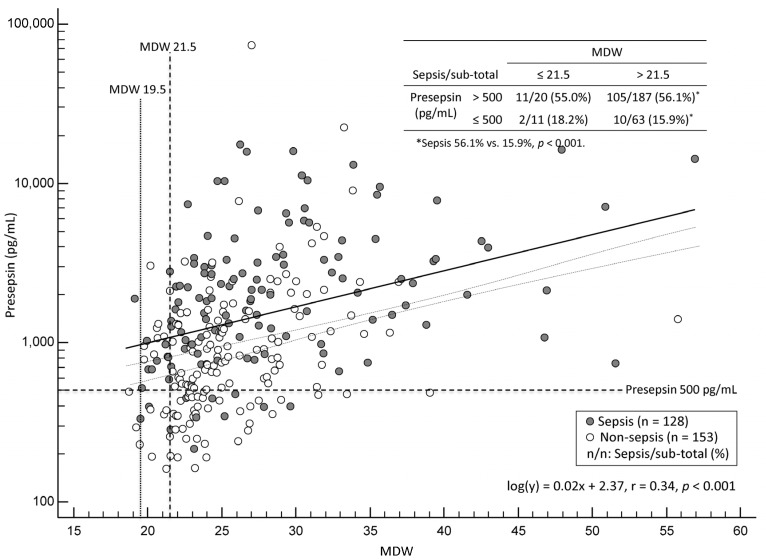
Scatter plot of monocyte distribution width (MDW) and presepsin levels in patients with sepsis (n = 128, black circles) and non-sepsis (n = 153, white circles). The presepsin axis is shown on a logarithmic scale. The plot is partitioned into quadrants using the cut-offs of MDW and presepsin.

Figure 1 shows the distribution of patients according to the MDW and log-transformed presepsin levels that were partitioned into the quadrant. A low correlation was observed between MDW and log-transformed presepsin levels (r = 0.34 [95% CI, 0.23–0.44], *p* < 0.001). In the patients with MDW above the manufacturer-suggested cut-off of 21.5, the proportion of sepsis was significantly higher in those with presepsin > 500 pg/mL than in those with presepsin ≤ 500 pg/mL (105/187 [56.1%] vs. 10/63 [15.9%], *p* < 0.001); no statistical difference was observed in the other quadrants. In the lower-left quadrant, where both MDW and presepsin levels were below their respective cut-offs, there were only two patients with sepsis (2/128, 1.6%). In these two patients, MDW values were 20.1 and 19.5, and presepsin levels were 397 pg/mL and 333 pg/mL, respectively; accordingly, 19.5 was set as the rule-in cut-off for MDW based on our data.

Table 3 shows diagnostic performances of MDW, presepsin, and their combination. MDW and presepsin showed sensitivities of 90.6% and 89.8%, respectively, and their combination improved the sensitivity up to 98.4% in diagnosing sepsis. The AUCs for MDW, presepsin, and their combination were comparable (0.52, 0.65, and 0.52, respectively), with no statistical difference.

**Figure 2:**
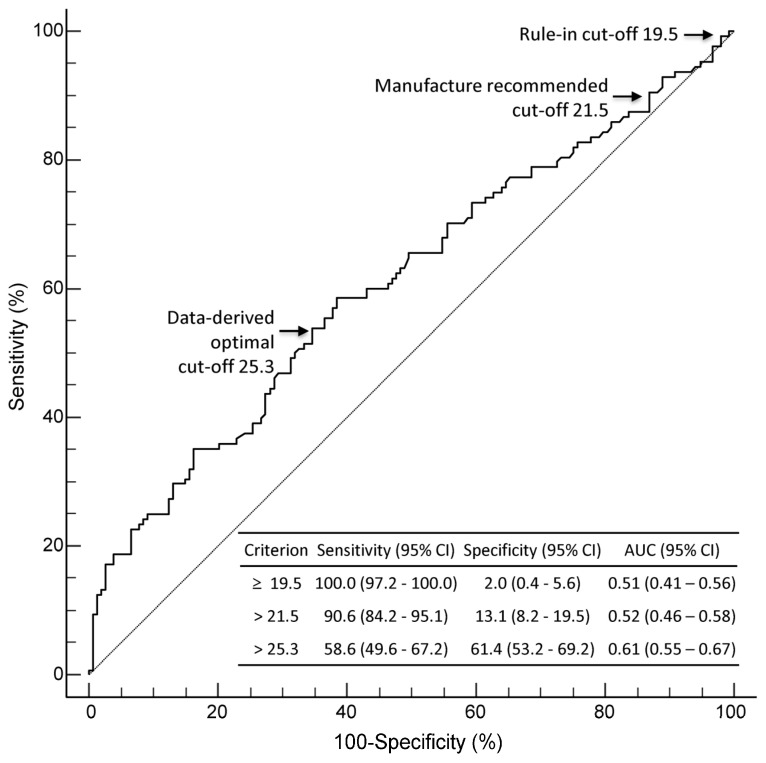
ROC curve analysis for MDW in diagnosing sepsis. Diagnostic performance of MDW was compared at three cut-offs: manufacturer-suggested cut-off (21.5), rule-in cut-off (19.5), and data-derived optimal cut-off (25.3). Abbreviations: AUC, area under the curve; CI, confidence interval; MDW, monocyte distribution width; ROC, receiver operating characteristic.

In the ROC curve analysis, the diagnostic performance of MDW was compared at three cut-offs: data-derived optimal cut-off (25.3), manufacturer-suggested cut-off (21.5), and rule-in cut-off (19.5, the lowest MDW value observed in confirmed sepsis patients) (Figure 2). The AUC of a data-derived optimal MDW cut-off of 25.3 was 0.61 (95% CI: 0.55–0.67), indicating moderate accuracy for sepsis diagnosis. The highest sensitivity of 100.0% was achieved using an MDW rule-in cut-off of 19.5; however, it showed a poor specificity of 2.0%.

**Figure 3:**
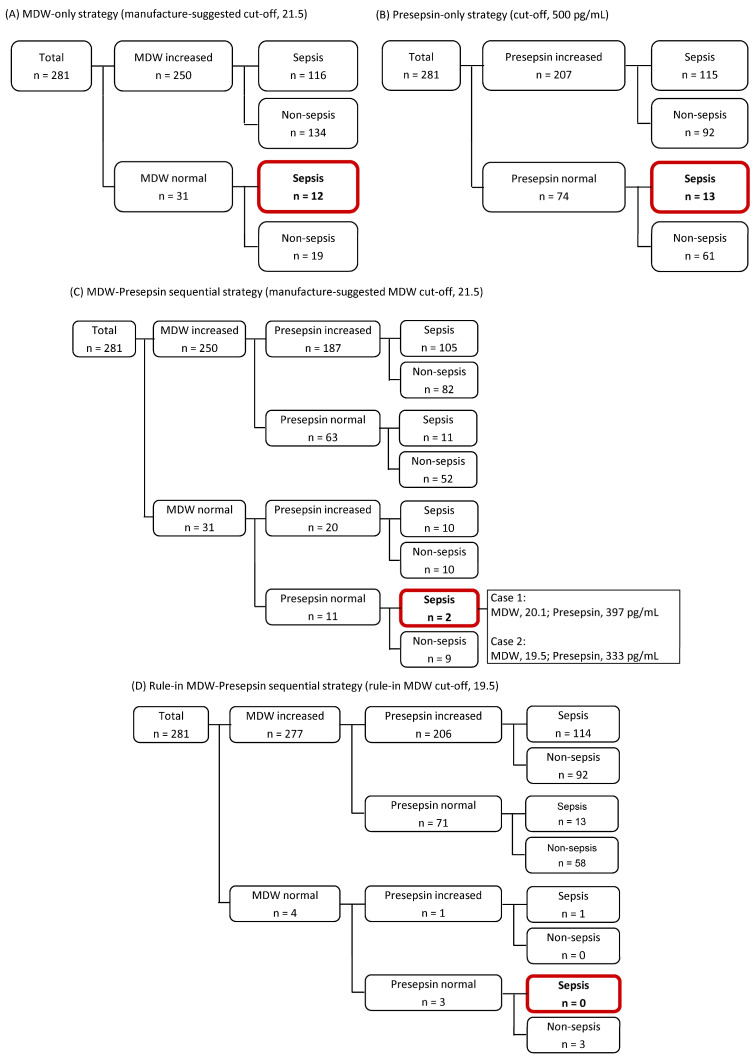
Diagnostic strategies for sepsis using monocyte distribution width (MDW) and presepsin. (**A**) MDW-only strategy using the manufacturer-suggested cut-off of 21.5. (**B**) Presepsin-only strategy using a cut-off of 500 pg/mL. (**C**) MDW–presepsin sequential strategy in which presepsin level was measured only when MDW was ≤21.5. (**D**) Rule-in MDW–presepsin sequential strategy using lowered MDW rule-in cut-off of 19.5.

Figure 3 shows diagnostic strategies for sepsis using MDW and presepsin. Using the manufacturer-suggested MDW cut-off of 21.5, 250 out of 281 patients (88.9%) showed an increased MDW value, while 31 patients (11.0%) showed a normal MDW value. Among the 128 septic patients, 12 (9.4%) had MDW values below the cut-off (Figure 3A). When presepsin was applied (cut-off of 500 pg/mL), 207 patients (73.7%) showed an increased presepsin level, and 74 patients (26.3%) showed a normal presepsin level. Among the 128 patients with sepsis, 13 (10.2%) showed presepsin levels below the cut-off (Figure 3B). When applying MDW–presepsin sequential strategy (measuring presepsin only when MDW was normal), using the MDW cut-off of 21.5, missed sepsis diagnoses were reduced to 2/128 (1.5%) compared with MDW alone (12/128, 9.4%) and presepsin alone (13/128, 10.2%) (*p* < 0.001; Figure 3C). When the MDW cut-off was lowered to the rule-in cut-off of 19.5, this rule-in MDW–presepsin sequential strategy could detect all sepsis patients, eliminating false negatives (Figure 3D).

## 4. Discussion

To our knowledge, this is the first study that explored a combined use of MDW and presepsin testing for early sepsis detection in adult patients in a real-world ED setting. In particular, we demonstrated that a sequential testing strategy of MDW and presepsin would be beneficial for triaging these critically ill patients in a timely and effective way. Using the previously suggested MDW cut-off, the sequential strategy could increase the diagnostic sensitivity, reducing false negatives, compared with either biomarker alone. Further, we applied a rule-in MDW cut-off to increase its sensitivity and eliminate all false negatives. This practical pathway would leverage MDW’s availability within the routine CBC to provide rapid first-pass triage, with presepsin being applied selectively to avoid missed sepsis [31].

The significantly higher median levels of both MDW and presepsin in the sepsis group compared to the non-sepsis group confirmed their diagnostic value in early sepsis detection (Table 1). These findings are in line with previous findings, reinforcing their roles as reliable early biomarkers of infection and systemic immune activation [14,16,25,32,33,34,35]. In our data, the Q1 values of MDW (22.8) and presepsin (494 pg/mL) approximated the designated or manufacturer-recommended cut-offs (MDW > 21.5 units; presepsin > 500 pg/mL), respectively [14,26,27]. Among the patients whose MDW and presepsin levels exceeded their Q1 values (Q2–Q4), the occurrence of sepsis was significantly higher, even when qSOFA was <2 (Table 2). This suggests that their respective cut-offs may reflect clinically meaningful thresholds that differentiate low- and high-risk patients of sepsis. The Sepsis-3 task force recognized that qSOFA excels in specificity rather than sensitivity, and it should not be used as a “rule-out” screening tool [1,31]. Considering that qSOFA may miss a meaningful subset of sepsis patients, incorporating MDW and presepsin could help detect qSOFA-negative sepsis patients and strengthen early sepsis detection in real-world ED settings.

MDW and log-transformed presepsin showed only a low correlation (r = 0.34, *p* < 0.001), indicating their distinct biological roles (Figure 1). MDW reflects morphological variability of monocytes (dispersion of monocyte size), whereas presepsin reflects monocyte activation and shedding of soluble CD14 in response to bacterial stimuli. This finding supports that each assay captures a distinct facet of the monocyte-derived immune response [36,37]. It also implies a limited redundancy between the two biomarkers as well as their complementary diagnostic utility. In our study, only two sepsis patients (1.6%) appeared in the quadrant where both MDW and presepsin levels were below their respective cut-offs; this may underscore the complementary value of the combined use of MDW and presepsin in effectively reducing false negatives (Figure 1). Although these two patients had biomarker levels below the cut-offs, their MDW and presepsin levels were still slightly elevated. This implies that even slightly abnormal values, though technically negative, may reflect early or borderline sepsis. Therefore, applying a lowered rule-in cut-off in clinical triage may be reasonable to reduce the risk of missed diagnoses.

Our findings confirm the clinical utility of MDW, presepsin, and their combined use as early sepsis detection biomarkers [2,6,38]. Each biomarker showed high sensitivity, and combining MDW with presepsin increased sensitivity to 98.4% (false-negative rate of 1.6%), which is beneficial in time-critical triage (Table 3). Although the AUCs for MDW and presepsin were modest (0.52 and 0.65, respectively), the high sensitivity highlights their utility as triage tools rather than definitive diagnostics [2,3]. On the other hand, our data also demonstrate that diagnostic accuracy remains low despite sensitivity improvements, and overinterpretation of the results should be avoided. Using an MDW rule-in cut-off of 19.5 further increased its sensitivity to 99.2% but decreased its specificity to 2.0%; this implies the role of MDW as an early sepsis detection tool, given its availability within the routine CBC (Figure 2).

Using an MDW cut-off of 21.5, 31 patients (11.0%) were considered negative, but 12 patients (38.7%) were later confirmed to have sepsis. This finding suggests that the FDA-cleared designated cut-off, while designed to identify patients at risk of developing sepsis within 12 h, may lack sufficient sensitivity in real-world ED settings. In our study, implementing a sequential MDW–presepsin strategy for MDW-negative patients reduced missed sepsis cases to just two out of 128 patients (1.6%). Moreover, applying the rule-in-MDW–presepsin strategy, using a rule-in MDW cut-off of 19.5, eliminated all false negatives, although this resulted in decreased specificity (Figure 3). In high-risk ED settings, lowering the MDW cut-off may improve early identification of sepsis, and the compromised specificity may be acknowledgeable to avoid missing critical cases. The right selection of the targeted patients is one of the key questions in the ED, and the quality and efficiency of patient care, including clinical decision-making, antibiotic administration, and ED resource utilization, are highly dependent on the strategic choice of laboratory testing [39]. Like high-sensitivity cardiac troponins in relation to myocardial injury, biomarkers with excellent sensitivity would be persuadable in stratifying ED patients with suspected sepsis [31,40].

In a previous meta-analysis, the overall diagnostic performance of MDW was comparable with that of PCT and CRP [31]. Although the pooled sensitivity of MDW was significantly higher than that of PCT, the pooled specificity was significantly lower than that of PCT. The specificity of a biomarker can be influenced by the pretest probability of a positive result; PCT is usually ordered for patients who are highly suspected of sepsis, although MDW is available for all ED patients. In this regard, the lower specificity of MDW can be explained by the lower pretest probability of sepsis. It has been known that presepsin expression is infection-specific compared with conventional biomarkers of PCT, CRP, and lactate, with a shorter response time after onset and shorter half-life in blood [41]. Although PCT has been explored in numerous studies, its clinical utility should be understood in the context of guiding antibiotic stewardship [42]. Moreover, the consensus on its utility is for antibiotic discontinuation, and single measurement would have limited value. Taken together, sequential application of MDW and presepsin would be a reasonable option in the ED setting.

This study has several limitations. First, it was a single-center retrospective study, which may limit the generalizability of the findings to other various settings with different patient populations or clinical practices. We relied on existing medical records, which may be incomplete or inconsistent, introducing potential information bias and limiting causal inference. Second, the cohort was restricted to adult ED patients, and other age groups (e.g., neonates, pediatrics, and the elderly) were not represented, introducing potential selection bias. This exclusion limits the applicability of our findings and should be considered in the context of real-world ED heterogeneity. In addition, the small number of patients with septic shock restricted our ability to perform a detailed stratified analysis, suggesting that future studies should focus on a larger cohort of septic shock patients to better understand the role of these biomarkers in disease severity. It is also essential to consider the specific characteristics of an ideal sepsis screening biomarker [31]. Crucially, the sequential strategy, despite achieving high sensitivity, was accompanied by extremely low specificity (5.9% and 2.0% with the rule-in cut-off). This low specificity directly compromises the goal of a cost-effective screening tool by increasing the risk of over-triage, unnecessary testing, and an additional resource burden in the ED. Presepsin’s non-universal availability further compounds this issue, requiring careful consideration of its impact on actual clinical management, such as antibiotic initiation and admission decisions. We explicitly acknowledge that optimal cut-off values are likely to vary across different populations, platforms, and clinical contexts. This is supported by a recent study on neonatal late-onset sepsis that proposed increasing the presepsin cut-off from 500 to approximately 713 pg/mL [43]. Accordingly, cut-off values, particularly the MDW rule-in cut-off, may be adjusted in accordance with various patient populations and clinical settings. Lastly, this study’s cross-sectional design lacked serial measurements, limiting the assessment of dynamic changes in MDW and presepsin over time, thereby limiting insight into their prognostic utility. Larger, multicenter, prospective studies are therefore needed to validate and expand our results, especially acknowledging how the findings may differ in those settings. Cost-effectiveness analyses and cut-off optimization in diverse patient groups such as pediatrics, neonatology, and geriatrics would be meaningful future research topics. Sepsis is a complex inflammatory pathophysiologic process; accordingly, integrating useful biomarkers into machine learning-based triage algorithms would increase their clinical utility [38,44].

In conclusion, this is the first study that evaluated the sequential strategy of MDW and presepsin for early sepsis detection in adult ED patients with suspected sepsis. MDW can be used as an initial testing, and then, presepsin can be used selectively as reflex testing. This sequential strategy would significantly improve diagnostic sensitivity and minimize false negatives compared with individual biomarker use. Moreover, our findings suggest that lowering the MDW cut-off, as a part of a rule-in sequential strategy, may further reduce missed sepsis cases. In spite of reduced specificity, this approach emphasizes a clinically acceptable compromise in time-sensitive triage scenarios. Integrating this sequential approach into routine clinical protocols would enhance early sepsis detection and improve patient outcomes. Prospective, multicenter studies are warranted to validate these findings and determine optimal rule-in cut-offs in various clinical situations.

## Figures and Tables

**Table 1:** Demographics, clinical, and laboratory characteristics of the study population.

Variable	Total (n = 281)	Non-Sepsis (n = 153)	Sepsis (n = 128)	*p*
Age (years)	68 (58–78)	65 (54–75)	72 (61–83)	<0.001
Males	172 (61.2)	88 (31.3)	84 (29.9)	0.76
Clinical outcomes				
ICU admission	77 (27.4)	29 (10.3)	48 (17.1)	<0.001
ICU stay (days)	3 (2–13)	2 (1–3)	5 (2–21)	0.002
Hospital stay (days)	16 (9–44)	12 (7–24)	29 (13–53)	<0.001
In-hospital mortality	43 (15.4)	10 (3.6)	33 (11.8)	<0.001
30-day mortality	38 (13.5)	9 (3.2)	29 (10.3)	<0.001
Comorbidities				
Cardiac diseases	18 (6.4)	12 (4.3)	6 (2.1)	0.283
Cerebrovascular accidents	117 (41.6)	50 (17.8)	67 (23.8)	<0.001
Chronic kidney diseases	25 (8.9)	12 (4.3)	13 (4.6)	0.498
DM/Metabolic syndromes	105 (37.4)	42 (15.0)	63 (22.4)	<0.001
Gastrointestinal diseases	50 (17.8)	18 (6.4)	32 (11.4)	0.004
Hematologic diseases	9 (3.2)	4 (1.4)	5 (1.8)	0.541
Hepatopancreatic diseases	22 (7.8)	14 (5.0)	8 (2.8)	0.145
Pulmonary diseases	76 (27.0)	28 (1.0)	48 (17.1)	<0.001
Solid cancers	31 (11.0)	17 (6.0)	14 (5.0)	0.963
qSOFA score	1 (1–2)	1 (1–1)	2 (1–2)	<0.001
SOFA score	2 (0–4)	1 (0–1)	4 (3–6)	<0.001
Cardiovascular	0 (0–0)	0 (0–0)	0 (0–2)	<0.001
Central nervous system	0 (0–1)	0 (0–0)	0 (0–2)	<0.001
Coagulation	0 (0–0)	0 (0–0)	0 (0–1)	<0.001
Liver	0 (0–0)	0 (0–0)	0 (0–1)	<0.001
Renal	0 (0–1)	0 (0–0)	0 (0–1)	<0.001
Respiratory	0 (0–1)	0 (0–0)	0 (0–1)	<0.001
Laboratory parameters				
CRP (mg/L)	15.8 (12.4–21.3)	15.8 (12.4–22.1)	15.8 (12.5–20.9)	0.688
Lactate (mmol/L)	1.82 (1.31–2.59)	1.61 (1.19–2.08)	2.05 (1.39–2.92)	<0.001
Procalcitonin (ng/mL)	0.59 (0.20–2.44)	0.28 (0.15–1.14)	0.79 (0.33–4.50)	<0.001
Hb (g/dL)	10.3 (9.0–11.9)	10.6 (9.7–12.7)	9.7 (8.6–11.0)	<0.001
WBC (×10^9^/L)	10.9 (8.0–14.2)	10.3 (7.6–13.8)	11.3 (8.6–15.4)	0.045
PLT (×10^9^/L)	219 (152–292)	245 (178–318)	187 (121–269)	<0.001
MDW	25.2 (22.8–29.2)	24.5 (22.6–28.3)	26.5 (23.2–31.5)	0.001
Presepsin (pg/mL)	878 (494–1660)	590 (377–1059)	1384 (776–2350)	<0.001

Data are presented as numbers (percentages) or median (interquartile range). Lactate levels were measured in 208 patients (non-sepsis, n = 89; sepsis, n = 119), and procalcitonin levels were measured in 206 patients (non-sepsis, n = 86; sepsis, n = 120). The ICU stay was counted for 77 patients admitted to the ICU. Abbreviations: CRP, C-reactive protein; DM, diabetes mellitus; Hb, hemoglobin; ICU, intensive care unit; MDW, monocyte distribution width; n, number; PLT, platelets; qSOFA, quick sequential organ failure assessment; SOFA, sequential organ failure assessment; WBC, white blood cells.

**Table 2:** Distribution of MDW and presepsin quartiles (Q1 vs. Q2–Q4) in the sepsis patients stratified by qSOFA.

	qSOFA < 2 (n = 184)		qSOFA ≥ 2 (n = 97)	
	Non-Sepsis (n = 129)	Sepsis (n = 55)	Non-Sepsis (n = 24)	Sepsis (n = 73)
MDW				
Q1 (n = 71)	37	15	7	12
Q2–Q4 (n = 210)	92	40	17	61
OR (95% CI)	4.2 (1.4–12.8)		8.3 (4.3–15.9)	
Presepsin				
Q1 (n = 73)	55	4	6	8
Q2–Q4 (n = 208)	74	51	18	65
OR (95% CI)	18.3 (4.2–79.5)		5.2 (2.8–9.9)	

Common ORs (95% CI) for MDW and presepsin were 7.0 (4.0–12.2) and 6.2 (3.4–11.0), respectively. No heterogeneity of strata was detected, and a common OR was estimated using the Cochran–Mantel–Haenszel (CMH) method. Abbreviations: CI, confidential interval; MDW, monocyte distribution width; n, number; OR, odds ratio; Q, quartile; qSOFA, quick sequential organ failure assessment.

**Table 3:** Diagnostic performance of MDW, presepsin, and combination of MDW and presepsin.

	Non-Sepsis (n = 153)	Sepsis (n = 128)	Sensitivity (%, 95% CI)	Specificity (%, 95% CI)	AUC (95% CI)	Accuracy (%, 95% CI)
MDW						
Increased (n = 250)	134	116	90.6 (84.2–95.1)	12.4 (7.7–18.7)	0.52 (0.46–0.58)	48.0 (42.1–54.1)
Normal (n = 31)	19	12				
Presepsin						
Increased (n = 207)	92	115	89.8 (83.3–94.5)	39.9 (32.1–48.1)	0.65 (0.59–0.70)	62.6 (56.7–68.3)
Normal (n = 74)	61	13				
MDW + Presepsin						
Increased (n = 270)	144	126	98.4 (94.5–99.8)	5.9 (2.7–10.9)	0.52 (0.46–0.58)	48.0 (42.1–54.1)
Normal (n = 11)	9	24				

The applied cut-off of MDW was 21.5. Abbreviations: AUC, area under the curve; CI, confidence interval; MDW, monocyte distribution width.

## Data Availability

The raw data supporting the conclusions of this article will be made available by the authors on request.
